# Supplementary material for: Association between oral microbiome and seven types of cancers in East Asian population: a two-sample Mendelian randomization analysis
Source: Front Mol Biosci. 2023 Nov 21;10:1327893. doi: 10.3389/fmolb.2023.1327893 (PMC10702768; doi:10.3389/fmolb.2023.1327893)
Supplement: Supplementary file 1 [file DataSheet1.ZIP › Supplementary Materials/MR plots of saliva/saliva═╝/Breast cancer/pheno.2481_to_breast cancer_forest.pdf]

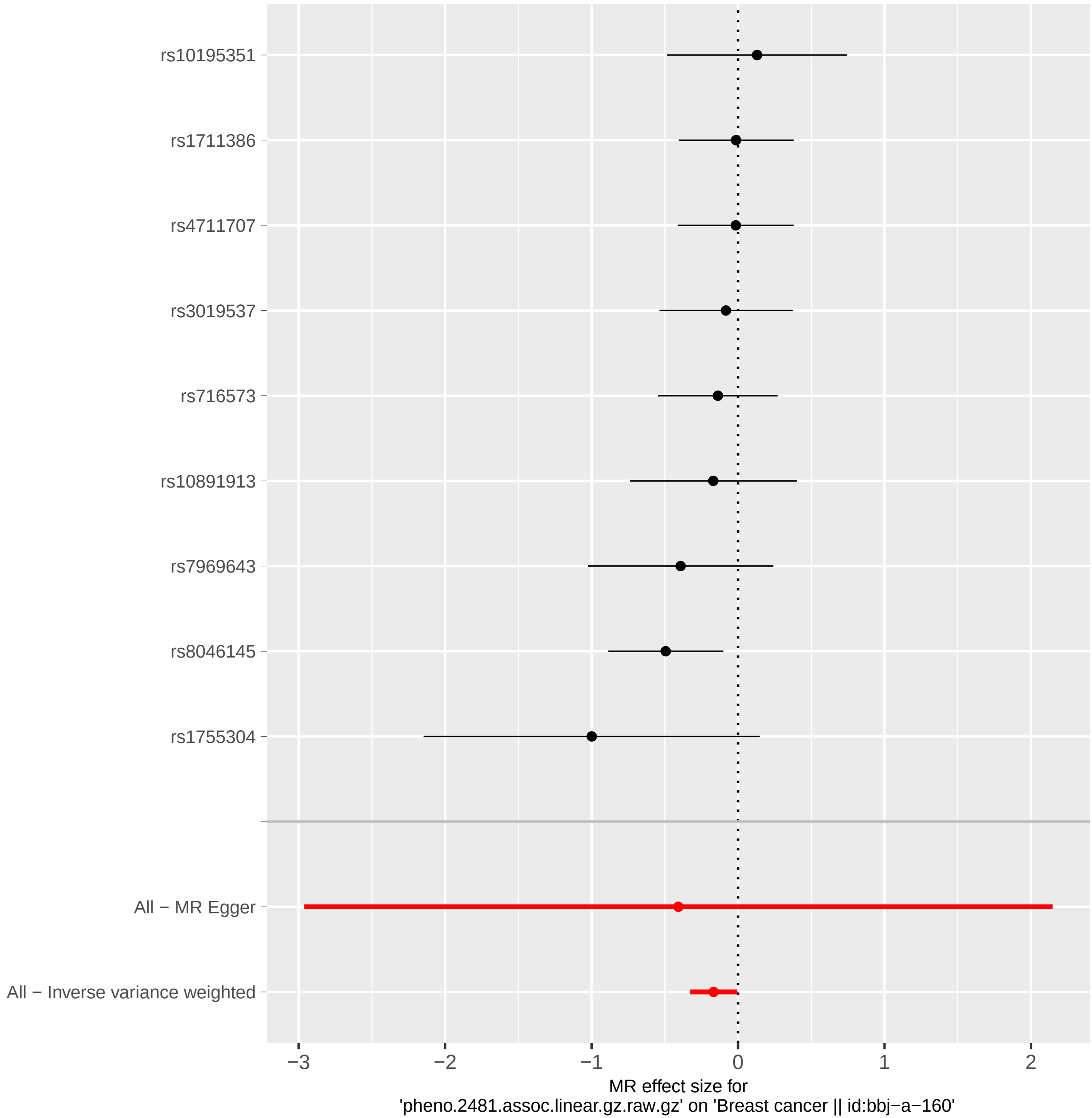
rs10195351
rs1711386
rs4711707
rs3019537
rs716573
rs10891913
rs7969643
rs8046145
rs1755304
All − MR Egger
All − Inverse variance weighted
−3
−2
−1
0
1
2
MR effect size for
'pheno.2481.assoc.linear.gz.raw.gz' on 'Breast cancer || id:bbj−a−160'
